# Uncertainty and precaution in hunting wolves twice in a year: Reanalysis of Treves and Louchouarn: Reply to Stauffer et al.

**DOI:** 10.1371/journal.pone.0319800

**Published:** 2025-03-25

**Authors:** Adrian Treves

**Affiliations:** Nelson Institute for Environmental Studies, University of Wisconsin, Madison, Wisconsin, United States of America; University of Bucharest, ROMANIA

## Abstract

Stauffer et al. (2024) present an alternative approach to modeling a one-year change in the wolf population of the state of Wisconsin, USA. They found an error in the code in Treves & Louchouarn 2022, which we corrected. It did not change that paper’s conclusions. However, Stauffer et al. accept the state of Wisconsin’s estimate for wolf abundance in 2022, which is based on undescribed methods, unshared data, lacks peer review, and depends on a method we have criticized for imprecision, inaccuracy, insensitivity to changing conditions, and irreproducibility. An occupancy model constructed and validated for a period several years after legal wolf-killing is a dubious basis for estimating wolf abundance one year after unprecedented, legal wolf-killing. Finally, undisclosed data continue to mar the work of state-funded scientists.

## Introduction

Stauffer et al. [1], hereafter S2024, criticized Treves & Louchouarn 2022 [2], hereafter TL2022, in which we attempted to fix a shortage of data during a policy process in Wisconsin. The policy process from 1 March–31 October 2021 resulted in the implementation of a second wolf-hunting season in one year that a state court halted (GLWA v WDNR 2021, Circuit Court Dane County, WI, Case 2021CV002103 Document 5). Late in October 2021, we concluded that even low quotas for a second public wolf-hunt in one year generated detectable probabilities of crossing undesirable legal thresholds for the wolf abundance statewide [2]. Although a state court order ended that planned wolf-hunt, TL2022 remained relevant because we had modeled the scenario of a zero-quota wolf-hunt to predict the state wolf population in April 2022. We used peer-reviewed data to simulate bounds of uncertainty about unmeasured or highly uncertain estimates of reproduction and survival to estimate a one-year change in wolf abundance. Note that estimating a one-year step change in wolf abundance can be modeled in several ways. S2024 propose another approach, but that does not mean we are wrong as S2024 suggested.

In contrast with S2024 which used state abundance estimates and assert that these are “actual” p.5, S2024 (i.e., real) data, we consider that their input data has serious shortcomings and other approaches provide a different picture of wolf population status. First, TL2022 began with a peer-reviewed estimate of the Wisconsin wolf population in April 2021 [3]. S2024 do not have a peer-reviewed estimate of wolf abundance for any of the relevant years 2020-2022. To counter their assertion of what is “actual”, I devote some text to explaining why the state abundance estimate has serious shortcomings (below).

TL2022 also made a good faith correction and an evaluation of an alternative life history parameter value, neither of which changed TL2022’s main conclusions [4]. S2024 did not cite our comment or our correction. I emphasize that the input data (wolf counts, mortality, birth estimates) deserve the most attention not the issue of which model one prefers for a one-year population change.

S2024 claim that TL2022 was (a) biased, in error, and incorrect in several passages; and their estimates are (b) correct, actual, and accurate in several assertions without evidence. However, S2024 found only one error, which was an arithmetic one we already acknowledged and corrected in [4]. They also claimed without sharing data or citing a peer-reviewed source that we should have used a different parameter value for reproduction, with which we disagreed [4].

### State estimate of wolf abundance

On pages 5-6, S2024 wrote, “The actual estimated spring 2022 population size, after realized zero harvest in fall 2021, was 972 (95% credible interval = 812–1,193) [8].” S2024 seem to believe their own estimates are “actual” truth. Their claim rests on citation 8 to “Wisconsin DNR. Wisconsin Gray Wolf Monitoring Report 15 April 2021 through 14 April 2022. Bureau of Wildlife Management. 2022”, which is not peer reviewed, does not contain even summary data on each survey and does not detail methods [5]. To understand why this is problematic, I need to review briefly the history of scientific debate over Wisconsin wolves.

### Scientific debate over Wisconsin wolf life history and abundance estimation

The state estimate of wolf abundance that S2024 prefer is a method that depends on annual winter snow-tracking, a method with the following shortcomings.

First, identification of wolf tracks in snow has not been subject to validation since 2000 and that validation by Wisconsin suggested substantial differences between state agency staff and civilian volunteers [6]. To this day, civilian volunteers conduct much of the wolf tracking in the snow. Counts of pack size done at this time and in a subsequent curation of such data, which has never been described in a peer-reviewed article, is verified for only a small percentage of wolf packs by aerial radio-telemetry (fewer than 13% of packs [7]. Therefore, most wolf packs in Wisconsin are identified by an imprecise and uncertain method without scientific accounting for the identity of the trackers or possible double-counting of the same wolves among other possible imprecisions [5]. Nor are all areas surveyed in this way every year as they once were [5].

Second, those input data on wolf presence have not been subject to peer review specific to wolf-counting methods, since the methods were altered in the period 2000-2004 [8]. During that period, we showed why estimates of pack size and estimates of pup survival to winter were confounded [9]. Raw data on wolf pack size and pup survival have never been published [10]; the summaries of such data only cover until 2007 [7]; and when models used those data, they neglected to include scripts, data, and clear figures [11]. Although on page 8, S2024 claim to have “extensive snow-tracking data”, those data are not presented in S2024 or any other peer-reviewed scientific journal.

Third, the method for abundance estimation raises additional concerns. The wolf presence data from mainly snow tracking, described above, are incorporated into a scaled occupancy model published by many of the same authors [12]. We have addressed inaccuracy, imprecision, insensitivity to changing conditions, and irreproducibility of the curation of wolf presence data and the model that uses those curated data in a prior paper [5]. Those concerns remain unanswered and will continue to be disputed until the state make the data fully and transparently available with detailed methods. This is not a new problem as we previously dissected how a lack of transparency in state wolf population data and models was causing problems for state claims [13].

Fourth, the 2024 state estimate of wolf abundance underpins the S2024 claims about quotas exceeding 300. S2024 presumes that Stauffer [12] had previously presented data or at least moves readers from summaries of data to final estimate. It does not as we have demonstrated in exhaustive detail [5]. Stauffer et al.’s scaled-occupancy model [12] was not validated for years following wolf-hunts [5]. The state implementation of that model does not seem to include a term for deduction of such deaths and explicitly risks counting dead wolves by using previous years of data on wolf census [5]. Therefore, the burden seems to fall on S2024 to show that the state counted hunted wolves, how they did so, and what the scientific justification was for using census data from prior years when an unprecedented February wolf-hunt with high mortality interrupted the 2021 census [5]. Similar concerns apply to the 2022 wolf abundance estimate because the scaled occupancy method relies on several prior years’ data. I note that S2024 did not make this plain. Therefore, I remain skeptical of the state estimates of abundance based on the scaled occupancy model [12], which S2024 relies upon and which we previously debunked [5].

Also, S2024 misunderstood our methods for the one-year step estimate of wolf population change in TL2022. I find it ironic that S2024 wrongly assumes TL2022 double-counted mortalities when the state estimate informed by [12] counts some dead wolves as alive. Regarding double-counting the wolf-hunt mortality, I suspect the confusion on their part came from this passage in TL2022,

“The state’s justification for interrupting the new census method before 14 April 2021, when it would have been terminated as in previous years…, was that the wolf-hunt of 22–24 February made accurate and precise data collection impossible. Therefore, the wolf population estimate derived from the new census method in 2021 lacked non-hunt mortality from 25 February to 14 April 2021, which is a season of high mortality from winter conditions and illegal killing historically.… We are not aware of any effort to correct the new census method estimate, therefore it seems to be a systematic over-estimate of N_2021_. Furthermore, the state did not provide bounds on N_2021_ but given the reported value (1195) of N_2021_ equaled the central tendency of N_2020_ (also 1195), we assume here the same bounds minus the 218 wolves killed legally in the February wolf-hunt, hence 977 (739–1355).” (Internal citations omitted, TL2022).

I believe S2024 misunderstood that we had deducted February 2021 wolf-hunt mortality from both population estimates (traditional and new scaled-occupancy-model approach), but we did not. TL2022 deducted those only from the new approach. We find no evidence that the new occupancy model by Stauffer et al. [12] accounted for wolf-hunt mortality. Given the wolf census of 2021 ended prematurely on the day before the wolf-hunt began, the state estimate of the wolf population could not have included data during and after the wolf-hunt and therefore seems to assign probabilities > 0 of occupancy by dead wolves across much of the state [14]. That seems like a serious flaw in the scaled occupancy model underpinning S2024’s population estimate; see [12] rebutted by [5].

### Wolf reproduction

S2024 also question our pup survival and birth rate parametrization. Contrary to their claim in that we, “…wrongly halved the number of pups that survived to November…” and “…counting harvested wolves twice among the dead” — our methods did neither. They might simply have misunderstood Eq.3 in TL2022 to represent the first half of the yea r when it actually represents the second half of the wolf-year. Only the second half of the wolf-year exposed pups to adult mortality hazards. For hazard from birth to November, we had already accounted for pup mortality, using data from [15]. The debate over [15] remains unresolved [16]. S2024 revive it without explaining to readers what basis they have for claiming that [7] provides a better estimate of pups reaching independence than that estimate given by [15]. That debate between former Wisconsin DNR staff and current ones should have been explained in S2024. Yet, the methods in [7] are generally considered imprecise and inaccurate compared to mark-recapture studies like that of [15]. Instead of sharing raw data and validated scientific methods, S2024 assert their correctness and rely on summary data through 2007 without scientific descriptions [7], which was published in a chapter of a book edited by two S2024 co-authors. Numerous peer-reviewed critiques have been published on Wisconsin population dynamics presented in that book [10,11,13].

### Adult wolf mortality

The debate over Wisconsin wolf mortality has also persisted because the state does not require its authors to share data transparently [17]. We modeled how such data on wolf deaths can be presented line by line [18]. Instead, S2024’s co-authors published yet another rebuttal without sharing data [19], and we had to rebut them again [17]. Without more, clearer data and scientific presentation of methods, the debate will never rise above its current, arid level.

S2024 cite [20], which in my view perpetuated an error in modeling vital rates that we described twice [18,21]. Although [22] corrected their estimates of hazard, that correction was incomplete as my colleagues demonstrated by treating collared wolf disappearances as an independent endpoint deserving more careful analysis of competing risks over time [23,24]. Those findings have been replicated three more times for different populations and policy periods [25–27]. S2024 does not fairly summarize our findings. Instead, they repeat an unsupported claim that cryptic poaching is rare, “…only minor adjustment was needed (i.e., annual mortality was 25% instead of 24%).” That claim is untenable as I explain next.

Rates of disappearance of radio-collared wolves in four US populations range from approximately 25–50% of all wolves collared. Variation seems to depend on the intensity of monitoring where the Mexican gray wolves and red wolves had lower rates of disappearance and more frequent monitoring whereas the less-monitored Wisconsin and Michigan populations had higher rates of disappearance [23–28]. S2024 and related work have not addressed the association between rates of disappearance of collars and timing of policy periods, nor why wolves experience rates of disappearance two to four times higher than other marked wildlife, which experience rates of disappearance of 6–13% [29–31]. Studies of collar failure do not reach the rates of disappearance seen among Wisconsin wolves [32]. Habib et al. [32] provided a possible maximum estimate of 13–14% for collar failures leading to disappearance. For further detail, see [17]. Instead of fair citation and addressing the substance of the debate, S2024 embraces models that fail to include inter-year variation in rates of legal wolf-killing, do not handle competing risks with state-of-the-art techniques from biomedical research on survival, and withhold data from readers and peer researchers [23].

S2024 claims about parametrization bias and errors are shown above to be mere disputes about differing estimates. Their claims that we double-counted are unsubstantiated and seem to reflect misunderstandings. Their arguments that we should use better model specifications stumble on issues of non-independence of data, data that are not shared, and disputes over how to model.

In conclusion, scientific debate is healthy when all sides share data transparently and disclose all methods and potential competing interests. Although inevitably science grapples with uncertainties and historical data cannot be validated in many cases, I do see a reason for optimism. The current method for estimating abundance of Wisconsin’s wolves can be improved, perhaps using the latest genomic techniques. Such methods applied by independent scientists could serve to test the 2025 state wolf population estimate and cast the current scientific debate in a clearer light.
